# The system, the resident, and the preceptor: a curricular approach to continuity of care training

**DOI:** 10.1007/s40037-021-00671-y

**Published:** 2021-06-11

**Authors:** Allyson Merbaum, Kulamakan Kulasegaram, Rebecca Stoller, Oshan Fernando, Risa Freeman

**Affiliations:** 1 North York General Hospital, Toronto, ON Canada; 2 Department of Family and Community Medicine, University of Toronto, Toronto, ON Canada; 3 The Wilson Centre, University of Toronto, Toronto, ON Canada

**Keywords:** Continuity of care, Curriculum design, Distributed medical education, Family medicine training

## Abstract

**Background:**

Continuity of care (CoC) is integral to the practice of comprehensive primary care, yet research in the area of CoC training in residency programs is limited. In light of distributed medical education and evolving accreditation standards, a rigorous understanding of the context and enablers contributing to CoC education must be considered in the design and delivery of residency training programs.

**Approach:**

At our preceptor-based community academic site, we developed a system—resident—preceptor (SRP) framework to explore factors that influence a resident’s perception regarding CoC, and established variables in each area to enhance learning. We then implemented a two-year educational SRP intervention (SRPI) to one cohort of residents and their preceptors to integrate critical education factors and align teaching of continuity of care within curricular goals.

**Evaluation:**

Evaluation of the intervention was based on resident interviews and faculty focus groups, and a qualitative phenomenological approach was used to analyze the data. While some factors identified are inherent to family medicine, the opportunity for reflection is a unique component to inculcate CoC learning.

**Reflection:**

The SRP innovation provides a unique framework to facilitate residents’ understanding and development of CoC competency. Our model can be applied to all residency programs, including traditional academic sites as well as distributed training sites, to enhance CoC education.

**Supplementary Information:**

The online version of this article (10.1007/s40037-021-00671-y) contains supplementary material, which is available to authorized users.

## Background and need for innovation

Continuity of care (CoC) is an integral component of several healthcare professions that provide primary care including nursing and midwifery, as well as pediatrics, internal medicine, and family medicine [1, 2]. Globally, there is renewed attention to CoC as its impact on the quality of care is increasingly recognized [3]. Hennen originally described four domains of CoC (chronological, interdisciplinary, geographical, and interpersonal) [4] and two additional domains (informational and family) were added later. These domains form the basis of the doctor-patient relationship and have led to a strong emphasis on CoC during family medicine training. For example, the College of Family Physicians of Canada, identified “continuity of patient care and education” as a key component of the Triple C (comprehensive, continuity of care, centred in family medicine) competency-based curriculum in 2011 [5].

There have been several interventions at the level of residency training aimed at improving patient continuity, including longitudinal rather than block programs and fixed as opposed to variable half-day clinics. However, the pedagogic rationales and effectiveness of these interventions in inculcating CoC remain unknown [6–13]. With many accreditation standards suggesting patient panels as an integral component of training and the reality of the expansion of distributed medical education outside of traditional academic health centres, a rigorous understanding of the context and enablers contributing to CoC education must be considered in the design and delivery of residency training programs [14]. Leaders in education have long debated the role of patient panels as these are not a curricular expectation at all institutions. Tensions between accreditation standards and the reality of how residency programs design and deliver authentic patient care learning experiences have not been rigorously explored.

The Department of Family Medicine at the University of Toronto (DFCM) is one of the largest departments of family medicine in North America. Residents spend 24 months training at one of 14 family medicine teaching sites in the greater Toronto area or in a rural stream. Some of the sites operate in hospital-based family medicine teaching units with residents responsible for small patient panels, while others are situated in community-based practices where residents train alongside their preceptors in an apprenticeship model.

North York General Hospital (NYGH) is a large suburban community teaching hospital in the DFCM system. It has provided a highly sought-after community-based training model for family medicine residents since 1985. Residents work alongside their preceptors in a model that allows for continuity of education (supervision, learning environment, and curriculum) and continuity of patient care.

## Goal of innovation

In response to questions about how CoC could be learned in a site where residents are situated in a preceptor-based learning experience, we implemented a novel innovation to make CoC an explicit learning goal as part of residency training at NYGH. In this paper, we describe our innovation and discuss evaluation data related to its implementation, experience, and initial outcomes.

## Steps taken for the development and implementation of the innovation

The first phase of planning for the innovation included a literature review regarding CoC in clinical practice as well as medical education. We then interviewed the cohort of residents exiting the program to explore their CoC learning experience, including enablers and barriers.

Based on both sets of data, we developed the system—resident—preceptor (SRP) model to categorize factors that influence a resident’s perception regarding CoC, and we established variables in each area that may enhance the CoC learning experience. For example, under the “system” heading, we identified that residents in all community practices required remote access to their clinic electronic medical records, facilitating access to the patient charts to review test results and to patients they are scheduled to see in clinic. In the “resident” domain we identified that residents needed consistent expectations regarding their role in the continuity experience, including prioritizing a consistent weekly family medicine half-day clinic and ensuring follow-up with their patients. With regard to family medicine “preceptors”, we identified the importance of balancing exposure to varied practice styles and populations while preserving education continuity. The preceptors were also encouraged to graduate the level of responsibility for patient care, label feedback specific to CoC, and provide residents the opportunity to follow patients in other settings.

We then conducted another literature review regarding interventions related to CoC in postgraduate medical training that guided the development of the second phase of the innovation. Our final framework derived from the literature used existing understandings of CoC in all of its dimensions [4, 5]. These elements informed our objectives and the design of the final two-year intervention. The intervention was entitled the system—resident—preceptor intervention (SRPI) and included CoC-oriented workshops for the residents and preceptors and two novel formative assessment tools: a reflective practice exercise (RPE) and a continuity of care iterative evaluation (CC-ITER). Questions for both the RPE and CC-ITER were mapped to Hennen’s domains of CoC (Table S1 in the Electronic Supplementary Material [ESM]).

The residents’ orientation workshop reviewed the foundational elements of “continuity of patient care and education”. The goal was to provide a framework for CoC which residents could apply in their clinical practice over the course of residency. The RPE was designed as a formal opportunity for residents to reflect upon and broaden their understanding of their CoC experience. It allowed for deliberate consideration of experiences in which they provided continuity or care. Residents were asked to complete the RPE at 6, 12, and 18 months of the residency, coinciding with biyearly progress reviews with the Site Director. The review of the RPE with the Site Director was meant to reinforce the importance of this unique element of our discipline. During these review meetings, barriers to CoC were identified and possible solutions discussed.

Faculty received a professional development workshop similar to the resident orientation session, but with a specific focus on setting expectations, labeling CoC, providing feedback, and transitioning responsibility. While the workshop introduced preceptors to opportunities to enhance their teaching of CoC to their residents, the CC-ITER, part of the novel innovation, was designed to provide faculty with an opportunity to assess their resident’s understanding of CoC, sense of responsibility, and competence. It served as a reminder and reinforced the importance of implicitly teaching and then assessing their resident’s participation in the CoC experience for their patients. Faculty were asked to complete the CC-ITER at 6, 12, and 18 months, which coincided with biyearly progress review meetings.

## Evaluation of innovation

Evaluation consisted of understanding the residents’ and faculty members’ experiences of the SRP innovation and their perceptions regarding the outcomes of their experience. This took place in the context of a larger study including another site on CoC teaching approaches, which received Research Ethics Board approval in 2015 (NYGH REB # 15-0022). The findings of that study have been submitted for publication [15]_._

All first-year NYGH residents in the urban stream (*N* = 12) and their preceptors (*N* = 27) from the 2015–2017 cohort were invited by email to participate in the evaluation process. A qualitative phenomenological approach was used to understand the lived experience of both members of the dyads. We developed an interview guide to explore resident’s understanding of and experience with CoC. We intentionally allowed residents to describe what CoC meant to them. The research assistant conducted individual resident interviews and faculty focus groups at 6, 12, and 18 months. The sessions were recorded, transcribed, anonymized, and entered into qualitative data analysis software (NVivo). All four members of the team independently coded transcripts at each phase and preliminary codes were identified. These codes were reviewed after each phase. Interpretations were reviewed and identified themes were discussed until consensus was reached. All interview data was included in the analysis. Eight residents (67%) participated in one or more interviews. Nine preceptors (33%) participated in one or more focus groups.

Through the qualitative interviews, information was gathered about the residents’ CoC experiences, gaps in their learning, and areas for potential improvement. The faculty focus groups aligned with the interviews by providing an opportunity to reflect on their residents’ behaviour around continuity and brainstorm enhancements for their teaching.

Tab. 1 summarizes the themes of our analysis.

**Table 1:** Resident and preceptor experiences of continuity of care (CoC)

	Resident Narrative	Preceptor Narrative
Opportunities for experiential and shared learning more instrumental than formal curriculum	*“I’ve had a lot of opportunities to experience it and I think that’s probably more valuable than sitting down in a lecture and listening about continuity”*	*“I don’t know if I [talk about it] explicitly but we do it just through our actions of trying to book, and basically following up with labs, consults, phone calls, things like that”*
Disease evolution and “wanting to know how the story goes”	*“You get feedback from subsequent visits that your clinical reasoning was correct or incorrect …. I think that the critical part to my learning in terms of integrating continuity of care was two-fold. One is sort of broadening my perspective on a person’s health problem and not having just that snapshot in time, but also developing the skills to manage a problem on an ongoing basis because that’s the key part that you need when you’re out in practice”*	*“So, I think, however, over the two years, you do need to see some patients over and over again, to learn how to follow up, to see the fruits of your labor, to understand what the outcomes are, what you ordered, and what the impact is”*
Productive struggle promotes authentic engagement in the continuity experience	*“Scheduling patients for clinic is a process that has to negotiate my schedule, the patient’s schedule, and their medical need for an appointment”*	*“It’s a little overwhelming for staff at a busy office with six physicians to remember which patient has seen the resident. So, what we try to do is when the patient is in the office and they need follow up, my resident will speak directly to the front staff and say, ‘please book Mr. Smith two weeks from today with me.’ So, we try to have her help facilitate it that way”*
Learning by example through preceptor role modeling	“*The preceptors with whom I have trained are all comprehensive family physicians and they see their role in the healthcare system and their duty to their patient as taking care of them across the full spectrum of illness. Dealing with both physical and mental health issues, dealing with social issues, dealing with the social determinants of health. You can’t deal with that stuff in just a one-off setting”*	*“And so, I think maybe they learn it, to a certain extent, by seeing the advantage that a doctor who has a long-standing relationship with a patient has, to be able to assess a particular complaint or connect with a patient and help them through some kind of difficulty”*
Creating opportunities for reflective practice leads to different professional growth and continuous learning patterns	“I *thought it was very useful to consider specific patient encounters, several weeks or months after the fact. I reflected differently on them than I did right at the start. In several cases, doing the reflective practice exercise made me realize how influential that patient encounter or series of encounters had been on what I’m doing now”*	

## Critical reflection

The evaluation of the SRP revealed the impact of the innovation on the understanding of continuity of care by residents situated in community preceptors’ offices and embedded in the full scope of practice.

We learned that the CoC orientation workshop for residents provided a framework that residents were able to apply in their clinical practice over the course of their residency. The RPE allowed for deliberate consideration of patient care experiences in which they had demonstrated continuity of care, and the subsequent discussion with the Site Director helped to reinforce the importance of this unique element of our discipline. During these review meetings, barriers to CoC were also identified and possible solutions discussed. There was significant faculty engagement in supporting optimal CoC provision in their learners, while preserving the authentic preceptor-based model that is highly valued in our context. While the faculty development workshop introduced preceptors to opportunities to enhance their teaching of continuity of care to their residents, the CC-ITER served as a reminder and reinforced the importance of implicitly teaching and then assessing their resident’s participation in the continuity experience for their patients. The focus groups allowed for discussion with colleagues about the practical application of these principles. Our results demonstrate the importance of the cycle of experiential learning followed by periodic reflection which aligns with common theories of efficacious experiential learning [16].

In our innovation, the addition of the explicit space for periodic reflection and exploration of the learning with the Site Director allowed for a unique understanding of the importance of CoC. The RPE reinforced that CoC goes beyond repeated patient visits, in line with the domains described by Hennen [4]. This is consistent with the study by Asgarova and colleagues in which reflection was described by learners as “one of the processes by which real learning occurred” [17]. Guided reflection in the periodic review process has also emerged as part of the coaching framework that is increasingly being adopted for residency programs across Canada [18].

The SRP framework allowed us to categorize relevant factors and target our educational opportunities regarding CoC, while preserving our authentic learning environment. While previous Canadian accreditation standards imposed the expectation of a patient panel for every resident, the current standards take a different approach. They now require that educational experiences provide opportunities for the development of continuity of care, are organized to facilitate responsibility for continuity of care, and that the learning environments enable residents to experience continuity with and responsibility for a group of patients [19]. This is very much in keeping with our SRP innovation that includes the RPE and CC-ITER tools and prompts residents to reflect on their role and responsibility in continuity of patient care. It also provides an opportunity for their preceptors to evaluate their sense of responsibility and competency in this domain.

Thus, the SRP innovation provides a unique framework to facilitate residents’ understanding and development of CoC competency. This brings evidence to the appropriate change in accreditation standards that now recognize the distributed preceptor-based model as an important option. It is relevant to note that our innovation moved beyond the traditional notion of continuity of patient care and provided an increased emphasis on continuity of education (learning environment, supervision, and curriculum) as essential components that allow for the graduated responsibility and independence expected across the trajectory of the competency-based family medicine residency [20].

### Limitations

We recognize that the small sample size of a single cohort of residents at one urban residency site is a limitation in the evaluation of our innovation. However, the longitudinal nature of following residents and their preceptors over a two-year period allowed for an understanding of how CoC learning evolves over time.

As findings from the evaluation emerged, it became apparent that the voice of the patient was missing as an important component of the continuity experience. We then recruited patients from the faculty members’ practices involved in residency training and conducted one-on-one interviews similar to those conducted with residents and teachers. However, as the role of the patient was only identified part way through the study, our assessment of this important component was limited. Future work in this area could be directed at better understanding the patient role in CoC education.

## Conclusion

As more residency programs move forward toward different models of distributed residency training, an understanding of the important curricular elements that we have identified in the SRP model will be critical to their residents’ acquisition of knowledge, skills, and positive attitudes regarding CoC.

## Supplementary Information

**Table S1** Questions for the RPE and CC-ITER mapped to Hennen’s domains of CoC
