# Supplementary material for: The system, the resident, and the preceptor: a curricular approach to continuity of care training
Source: Perspect Med Educ. 2021 Jun 11;11(5):295–9. doi: 10.1007/s40037-021-00671-y (PMC9582181; doi:10.1007/s40037-021-00671-y)
Supplement: Supplementary file 1 — Table S1 Questions for the RPE and CC-ITER mapped to Hennen’s domains of CoC [file 40037_2021_671_MOESM1_ESM.docx]

**Table S1** Questions for the RPE and CC-ITER mapped to Hennen’s domains of CoC

|  | Reflective Practice Exercise | CC-ITER |
| --- | --- | --- |
|  | Choose three patients that you feel you demonstrated continuity in the previous 6 months. For each patient, please answer the following questions: | On a scale of 1-5 where 1 is not at all often and 5 is very often, how often does your resident take the opportunity to: |
| Chronologic | How many times did you see this patient? | Phone the patient or book the patient back in the office for follow up to review? |
| Geographical | Did you provide care outside of the office? In what setting? | See patients in other settings outside the office? |
| Informational | Did you order investigations? Did you review the results? Did you convey the results to the patient? | Review lab investigations?  Discuss the results of these investigations and their management? |
| Interdisciplinary | Did you involve other health care providers? | Communicate with the specialist or AHP or review the consult note?  Complete a referral to a specialist or AHP? |
| Family | Did you interact with other family members? |  |
|  | In what way did the doctor-patient relationship contribute to the management of the patient? |  |
|  | How did the experience contribute to your learning? Based on this experience, what will you do differently next time? What will you do the same? | Overall, to what degree does your resident display an interest or take responsibility for continuity of care with their patients? |
|  | Would you say this experience is something you will see again in future practice? |  |
